# Progressing left-side sciatica revealing a common iliac artery mycotic aneurysm in an elderly patient: A CARE-compliant case report

**DOI:** 10.1097/MD.0000000000022476

**Published:** 2020-10-09

**Authors:** Tzu-Yen Huang, Chi-Hsiao Yeh, Yao-Chang Wang, Yu-Ting Cheng, Pin-Chao Feng

**Affiliations:** a Department of Thoracic and Cardiovascular Surgery, Chang Gung Memorial Hospital at Keelung; b Department of Biomedical Engineering, National Taiwan University; c Department of Thoracic and Cardiovascular Surgery, Chang Gung Memorial Hospital at Linkou; d College of Medicine, Chang Gung University, Taiwan.

**Keywords:** endovascular repair, iliac artery aneurysm, iliac bifurcation stent graft, mycotic aneurysm, sciatica

## Abstract

**Rationale:**

Sciatica is usually caused by lumbar spine disease; the incidence of sciatica from extra-spinal causes is noted to be only about 0.09%.

**Patient concerns:**

We report a case of a 92-year-old man who came to the neurologist outpatient department due to left buttock pain and numbness that radiated to the left lower leg in the recent 6 months and progressed rapidly over 10 days.

**Diagnosis:**

We arranged magnetic resonance imaging for lumbar nerve lesion. Magnetic resonance imaging showed a common iliac artery mycotic aneurysm, at about 6.3 cm in diameter, which compressed the psoas muscle, nerve plexus, and vein.

**Interventions:**

We used a left-side iliac bifurcation stent graft of 12 mm in diameter for aneurysm repair. An internal iliac artery with a stent graft of 10 mm x 5 cm. An abdomen aortic aneurysm stent was inserted, 1 cm beneath the right renal artery from the right side femoral artery.

**Outcomes:**

After endovascular repair and 4 weeks of antibiotic treatment, he could walk again, and no sciatica was noted. We repeated computed tomography 5 months after the operation and noted that the size of the iliac artery aneurysm decreased without stent graft migration or extravasation. Our patient recovered from sciatic and left leg weakness; above all, he could walk again.

**Lessons:**

We suggest practitioners check for common iliac artery aneurysms in the diagnosis of symptoms mimicking spinal cord origin sciatica, especially in elder patients.

## 1 Introduction

Sciatica symptoms were first recorded by ancient Greek and Roman physicians and were well described in 1764 by Dr. Domenico Cotugno, who defined sciatica as a neurological symptom instead of being of arthritic origin.^[1]^ Sciatica is often the result of external compression, of which the most common causes are inter-vertebral disk rupture, spinal foraminal stenosis, central disk herniation, and spondylolisthesis.^[2]^ Sometimes, the external compression is caused by pelvic tumor. We report an atypical case of sciatica revealing a giant mycotic common iliac artery aneurysm (IAA). Patient has signed informed consent for publication of the anonymous case report.

## 2 Case report

We describe a case of a 92-year-old man with a history of angina, heart failure, and chronic obstructive pulmonary disease. He came to the neurologist outpatient-department due to left buttock pain and numbness that radiated to the left lower leg over 6 months and progressed rapidly over 10 days. The pain was also accompanied with muscle weakness, and he had difficulty walking.

**Figure 1:**
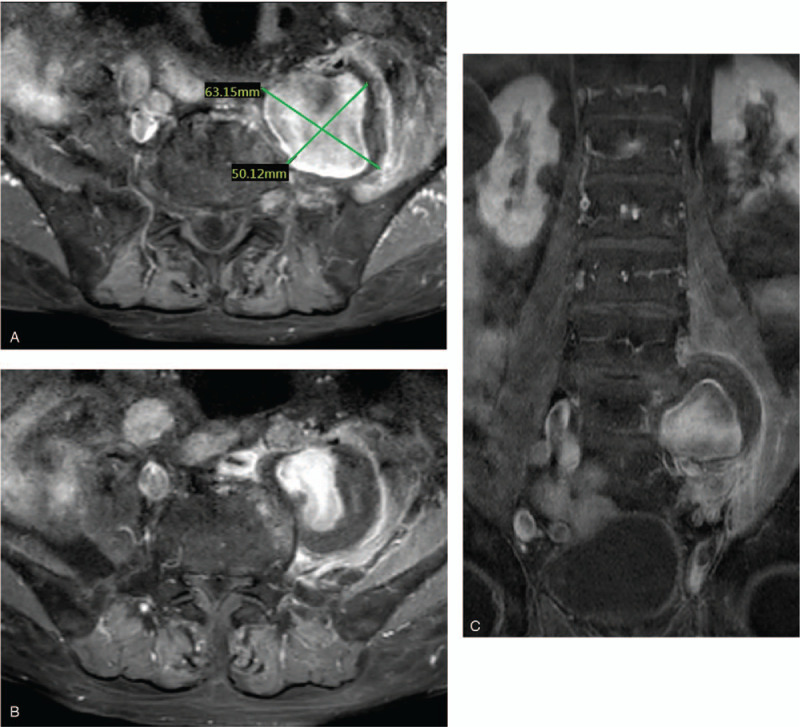
Magnetic resonance imaging (MRI) exam for spinal cord lesion: (A) a common iliac artery aneurysm compressed the psoas muscle, at 63 mm in diameter. (B) The aneurysm was from common iliac. (C) The aneurysm was fusiform; the internal and external iliac artery were patent without aneurysm changes.

Under the impression of lumbar spinal stenosis, he was admitted for a magnetic resonance imaging (MRI) exam for surgical intervention. The MRI exam showed spinal lesions, lumbar spondylosis with multi-level mild to moderate stenosis of lateral recesses, and neuroforamens. However, a common IAA of about 63 mm was noted just above the bifurcation of the external iliac artery and internal iliac artery; the aneurysm compressed the common iliac vein, local psoas muscle, and soft tissue (Fig. 1). A blood culture was taken showing Salmonella serogroup D without resistance to antibiotics. Under the impression of a mycotic aneurysm, an antibiotic, Ceftriaxone, was used before operation for mycotic common IAA. He was transferred to our cardiovascular department.

**Figure 2:**
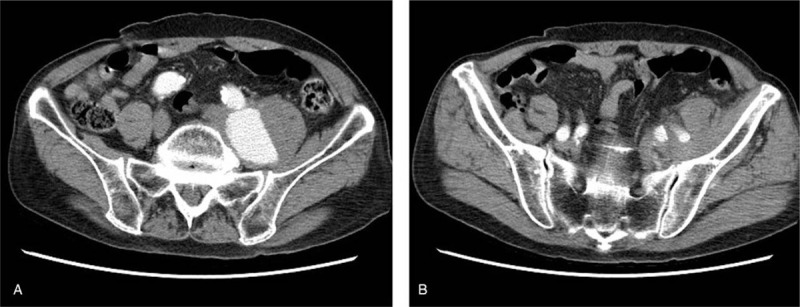
Computed tomography angiography (CTA) exam for pre-operation aorta evaluation. (A) Common iliac aneurysm with severe arterial calcification; (B) normal size of left internal iliac artery and external iliac artery.

We rechecked the patient's left leg condition, swelling, edema, and numbness from the left hip to toes without sensory loss. A left-side dropped foot was also noted, with decreasing muscle power of his left leg. We arranged a computed tomography angiography (CTA) of the aorta for pre-operation survey. The CTA showed an isolated common IAA without other aorta lesions (Fig. 2). After discussion with the patient and his family, we arranged an endovascular stent graft insertion for common IAA repair.

**Figure 3:**
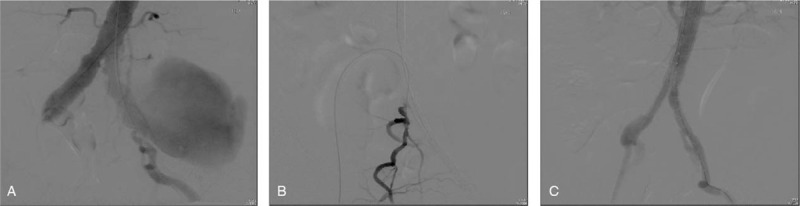
Intra-operation angiogram: (A) angiogram of the left common iliac artery aneurysm, (B) guiding the catheter wiring into the left internal iliac artery for the iliac bifurcation stent system. (C) After iliac bifurcation stent graft and abdominal aorta stent graft insertion, the aneurysm was excluded over the stent.

We used the ultrasound sonogram guide puncture method for bilateral common femoral artery for sheath insertion with 2 vessel closure devices (Abbott, Perclose ProGlide, Chicago, IL, USA) for each femoral artery. Angiography was done in the operation room and checked by a marked-pigtail. We used a left-side iliac bifurcation stent graft (IBD) of 12 mm in diameter for aneurysm repair (Cook medical, Zenith Branch stent, Bloomington, IN). An internal iliac artery with a stent graft of 10 mm x 5 cm (W. L. Gore, Viabahn, Newark, DE) was inserted and deployed. An abdomen aortic aneurysm stent was inserted from the right side, and the abdomen aortic aneurysm landing zone was about 1 cm beneath the right renal artery (Fig. 3). Bilateral femoral vessel wounds were closed by vessel closure devices.

**Figure 4:**
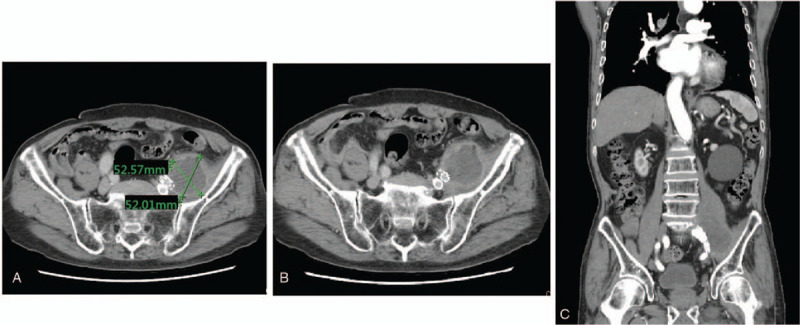
Five months after endo-vascular operation: (A) the diameter of the iliac aneurysm decreased without contrast extravasation. (B) Patent internal iliac artery and external iliac artery without endo-leakage. (C) Decreasing size of iliac artery aneurysm, with a coronal section view.

Left leg pain and numbness were much relieved and the lower-leg edema was much improved on the day after the operation. His muscle power of his left lower extremity mildly improved, and he could walk with a helper on the third day after the operation. Due to the impression of mycotic aneurysm, he received antibiotic treatment for 4 weeks and was then discharged. The patient was followed by the outpatient-department and maintained his rehabilitation course. A post-operation CTA followed 5 months later, which showed no contrast extravasation and a decreasing size of the IAA (Fig. 4). The patient could walk better and had no recurrent pain, numbness, or weakness of his left lower extremity. This study was approved by Chang Gung Memorial Hospital. Patient has provided informed consent for publication of the case.

## 3 Discussion

Sciatica is usually caused by lumbar spine disease; the incidence of sciatica from extra-spinal causes is noted at only about 0.09%.^[3–5]^ It is a rare condition and is easily neglected. When symptoms combined with lower extremity edema (venous compression) or flank soreness with knocking pain (hydronephrosis caused by ureter compression) are noticed, we suggest considering that sciatica may be caused by extra-spinal mass compression. Nevertheless, image studies of the spine could also discover the artery anatomy. There are some studies reporting similar conditions of IAA causing sciatica.^[6–10]^

A normal common iliac artery size is about 15∼18 mm, and IAA is defined as 1.5 times the artery diameter.^[11]^ The risk factors of an isolated iliac artery are old age (the seventh to the eighth decade), male (90% of cases), atherosclerosis, infection (mycotic), vasculitis diseases, and spontaneous dissection, as with aortic aneurysms.^[12,13]^ Some studies have also showed that IAA is caused by post-operation vessel injuries.^[14]^ In our case, the patient was a 92-year-old man with Salmonella infection, which matched 3 risk factors, plus the size of the aneurysm was 6.3 cm in diameter. Most IAAs are incidentally found by image studies which were not planned for an artery aneurysm, often because they are asymptomatic.^[11]^ In our case, the left common IAA compressed the lumbosacral plexus in the psoas muscle. The symptoms mimicked spinal cord compressions, which led the neurological department to arrange an MRI for a herniated intervertebral disk or spinal stenosis.

The IAAs are classified by different systems. Reber's classification defines different anatomical structures,^[15]^ while Fahrni's classification depends on the aneurysm neck for endovascular repair.^[16]^ Our case is Reber's Type I and Type Ib in Fahrni's classification. Most IAAs are considered to be associated with a high risk of rupture if the diameter is over 3.5 cm; surgical management is indicated to prevent aneurysm rupture.^[13]^ The indications for IAA surgery are aneurysm-caused symptoms, such as thrombus formation, mass effect (compression to ureter, nerve, or vein), severe abdominal pain due to aneurysm, or aneurysm rupture. In our case, the patient's clinical symptoms were caused by IAA compression, and the size of the aneurysm was much over 3.5 cm; therefore, surgery was the standard management.

The surgical treatment of IAAs is performed mainly to exclude the aneurysm from circulation in order to prevent further growth of the aneurysm and aneurysm rupture.^[13]^ The traditional surgery is laparotomy and to expose the aneurysm with an aneurysm resection or bypass. However, the operative mortality rate for elective open repair is as high as 10%.^[17,18]^ The endovascular stent graft technique has been well developed over the last 20 years and exhibits a significant decreased operation mortality and morbidity rate when compared with open repair.^[19–22]^ Endovascular repair may be considered to be the first-line therapy for patients with IAA.^[13]^ In mycotic aneurysm, endovascular repair is also an acceptable alternative to open repair.^[23,24]^

Our patient was a 92-year-old man with heart failure history. Therefore, we adopted endovascular operation as it could decrease the operation time, blood loss, morbidity, and mortality rate. Moreover, we used an IBD system for this patient because his aneurysm was located just above the bifurcation of the internal iliac artery and external iliac artery. The bifurcation system could not only preserve the blood perfusion of the internal iliac artery but also prevent type II endo-leakage from the retrograde flow of the internal iliac artery; a low complication rate and high technical success rate were also noted in other research.^[25–27]^

No studies have specifically reported follow-up guidelines in patients who underwent IAA repair surgery. Abdominal aorta aneurysm guidelines recommend the use of image data to evaluate an aneurysm's diameter, such as ultra sound, as well as the use of follow-up CTA every 5 years to evaluate long-term outcomes.^[13]^ We checked the CTA of this patient at 5 months after the operation. The size of the aneurysm decreased without clinical symptoms. An annual image examination is arranged.

## 4 Conclusions

IAA-caused sciatica is easy to neglect due to its low incidence. It should be checked if there are ureter or vein compression symptoms, especially in elderly patients. The taking of a complete history and physical examinations are very important. CTA helps in the diagnosis of IAA; however, a blood culture and infection survey should also be completed to rule out mycotic aneurysm. If mycotic aneurysm is found, at least 4 weeks of antibiotics treatment are required. In respect of surgical treatments in IAA, endovascular repair could be the first option, whether the aneurysm is mycotic or not. An IBD system would be a good choice due to the low complication rate. Sciatic symptoms would improve after IAA repair. Surgical intervention is beneficial to patients with symptomatic iliac aneurysms, even to our 92-year-old patient. Our patient recovered from sciatic and left leg weakness; above all, he could walk again. An annual image follow-up should be arranged to evaluate further changes of the aneurysm.

## Author contributions

**Conceptualization**: Tzu-Yen Huang, Chi-Hsiao Yeh.

**Investigation**: Tzu-Yen Huang, Yao-Chang Wang.

**Figures**: Yu-Ting Cheng.

**Supervision**: Tzu-Yen Huang, Chi-Hsiao Yeh.

**Visualization**: Yu-Ting Cheng, Pin-Chao Feng.

**Writing – original draft**: Tzu-Yen Huang.

**Writing – review & editing**: Tzu-Yen Huang, Pin-Chao Feng.

## Abbreviations

Abbreviations: CTA = computed tomography angiography, IAA = iliac artery aneurysm, IBD = iliac bifurcation stent graft, MRI = magnetic resonance imaging.
